# AdipoRon Affects Cell Cycle Progression and Inhibits Proliferation in Human Osteosarcoma Cells

**DOI:** 10.1155/2020/7262479

**Published:** 2020-01-22

**Authors:** Luigi Sapio, Ersilia Nigro, Angela Ragone, Alessia Salzillo, Michela Illiano, Annamaria Spina, Rita Polito, Aurora Daniele, Silvio Naviglio

**Affiliations:** ^1^Dipartimento di Medicina di Precisione, Università degli Studi della Campania “Luigi Vanvitelli”, Via L. De Crecchio 7, Naples 80138, Italy; ^2^Dipartimento di Scienze e Tecnologie Ambientali Biologiche Farmaceutiche, Università degli Studi della Campania “Luigi Vanvitelli”, Via G. Vivaldi 42, Caserta 81100, Italy; ^3^CEINGE-Biotecnologie Avanzate Scarl, Via G. Salvatore 486, Naples 80145, Italy

## Abstract

AdipoRon (AdipoR) is the first synthetic molecule acting as a selective and potent adiponectin receptor agonist. Recently, the possible pharmacological use of AdipoR in different pathological conditions has been addressed. Interestingly, initial evidence suggests that AdipoR may have anticancer properties in different preclinical models, such as pancreatic and ovarian cancer. To our knowledge, so far no research has been directed at determining the impact of AdipoR on osteosarcoma, the most aggressive and metastatic bone malignancy occurring in childhood and adolescence age. Here, we investigate the possible antitumor effects of AdipoR in osteosarcoma cell lines. MTT and cell growth curve assays clearly indicate that AdipoR inhibits, at different extents, proliferation in both U2OS and Saos-2 osteosarcoma cell lines, the latter being more sensitive. Moreover, flow cytometry-based assays point out a significant G0/G1 phase accumulation and a contemporary S phase decrease in response to AdipoR. Consistent with the different sensitivity, a strong subG1 appearance in Saos-2 after 48 and 72 hours of treatment is also observed. The investigation of the molecular mechanisms highlights a common and initial ERK1/2 activation in response to AdipoR in both Saos-2 and U2OS cells. Interestingly, a simultaneous and dramatic downregulation of p70S6K phosphorylation, one of the main targets of mTORC1 pathway, has also been observed in AdipoR-treated Saos-2, but not in U2OS cells. Importantly, a strengthening of AdipoR-induced effects was reported upon everolimus-mediated mTORC1 perturbation in U2OS cells. In conclusion, our findings provide initial evidence of AdipoR as an anticancer molecule differently affecting various signaling pathways involved in cell cycle and cell death in osteosarcoma cells and encourage the design of future studies to further understand its pattern of activities.

## 1. Introduction

Originating from primitive bone cells of mesenchymal derivation, osteosarcoma (OS) represents one of the most frequent primary malignancies that occur in childhood and adolescence [1]. The combination of a rapid and a high growth potential that marks bone cells in these two specific life stages may encourage the onset of the illness, defining both sites of occurrence, lower metaphysis of long bones and peak incidence, between 10 and 14 years [2]. Although OS etiology remains mostly unknown, two distinct genetic alterations are proposed to be closely related to OS predisposition, in particular, hereditary mutation of retinoblastoma and autosomic recessive mutation of p53 in Li-Fraumeni syndrome [3]. Distant macroscopic metastases can exist at the time of diagnosis in around 10–20% of OS patients, whereas for the remaining patients, subclinical or undetectable micrometastases are assumed to be present [4]. The lung is generally the elective substrate for OS metastasis, even if metastases can also occur in bone sites different from the primary tumor and rarely in lymph nodes [5]. As a highly aggressive sarcoma, prognosis for OS cases remains extremely poor and 5-year survival is extremely variable depending on the tumor stage and site of possible recurrence. Currently, 5-year event-free survival (EFS) is about 60–70% in localized and nonmetastatic disease, whereas in patients with pulmonary dissemination, EFS turns out to be 18–33% [6]. Complete surgical resection, preceded and followed by chemotherapy treatment, remains the unique and only partially effective approach for osteosarcoma cure [7]. Although the survival rate in OS patients is gradually increased in the last years in response to the multidrug treatment, more effective therapeutic modalities for OS treatment are absolutely needed [8].

Adiponectin (Acrp30) represents the most abundant circulating cytokine present in human serum [9]. Synthesized by adipocytes as a single monomer, Acrp30 is consecutively multimerized and secreted in three different forms: low molecular weight (LMW), medium molecular weight (MMW), and high molecular weight (HMW) [10]. The Acrp30 biological properties are mainly exerted through the interaction with seven-transmembrane receptors, ADIPOR1 and ADIPOR2 [11]. Moreover, an additional Acrp30 receptor, named cadherin13 (CAD13), has recently been discovered to have a high affinity for HMW species [12]. Several studies have clearly demonstrated the multivalent action of Acrp30 in several biological systems, including human, as inflammatory response modulator, energy homeostasis regulator, and insulin sensitizer [13–15]. In this regard, adiponectin levels are generally decreased in obese, insulin-resistant, and type 2 diabetes patients [16]. Extensive research has also brought to light the physiological role of Acrp30 in bone metabolism [17]. In particular, analyzing Acrp30 endocrine effects on the skeletal system in women, an inverse correlation between its serum concentration and bone mineral density (BMD) has clearly been demonstrated in different clinical studies [18, 19]. Conversely, the relative risk of developing bone fractures is positively associated to circulating adiponectin levels, especially in men [20]. Regarding Acrp30-mediated activity in osteoblast-like cells, contrasting results have been obtained in *in vivo* and *in vitro* preclinical models on proliferation; instead, more consistent data have been gained about the positive effects of Acrp30-induced osteoblast differentiation [17].

AdipoRon (AdipoR) is the first oral adiponectin receptor agonist capable of binding and activating AdipoR1 and AdipoR2 [21]. Over the past few years, AdipoR is emerging as a possible candidate for the treatment of different pathological conditions, including metabolic, cardiovascular, and psychiatric disorders, specifically comorbidity between depression and obesity [22–24]. According to the antitumor effects observed in response to Acrp30 [25, 26] and the opposite relation between its circulating levels and risk of developing cancer [27], initial reports have also investigated the possible anticancer role of AdipoRon in preclinical models, especially in pancreatic and ovarian cancer [28–30]. To our knowledge, no evidence has been published yet concerning the possible antiproliferative properties of AdipoR and more in general of Acrp30 in OS. For the abovementioned reasons, the current study has been designed to investigate the possible consequences of AdipoR on the cell viability, cell growth, and cell cycle progression in two different osteosarcoma cell lines (Saos-2 and U2OS) and on the underlying molecular mechanisms.

## 2. Materials and Methods

### 2.1. Chemical Reagents

Bovine serum albumin (BSA) (Microtech, #B2518), 3-(4,5-dimethylthiazol-2-yl)-2,5-diphenyltetrazolium bromide (MTT) (Sigma Life Science), propidium iodide (PI) (Sigma Life Science, #P4864), AdipoRon (Focus Bioscience, St Lucia, QLD, Australia), and everolimus (Cell Signaling Technology, #12017).

### 2.2. Antibodies

Anti-AdipoR1 (C-14) (#46748) and Anti-AdipoR2 (C-12) (#46751) were obtained from Santa Cruz Biotechnology. Anti-p44/42 MAPK (ERK1/2) (#9102), Anti-phospho-p44/42 MAPK (ERK1/2) (Thr202/Tyr204) (#9101), Anti-p70S6K (#9202), Anti-phospho-p70S6 Kinase (Thr389) (#9205), and Anti-GAPDH (14C10) (#2118) were purchased from Cell Signaling Technology. Anti-Vinculin (#13007) and Anti-Cadherin13 (#36905) were acquired from Abcam. Secondary horseradish peroxidase- (HRP-) conjugated antibodies were used for immunoblotting: goat anti-rabbit (GtxRb-003-DHRPX) and goat anti-mouse (GtxMu-003-EHRPX.0.05) (ImmunoReagents Inc.).

### 2.3. Cell Culture

Human osteosarcoma cell lines, U2OS and Saos-2, were obtained from the American Type Culture Collection (ATCC). Maintained at 37°C in 5% CO_2_-humidified atmosphere, cells were grown in Dulbecco's modified eagle's medium (DMEM) (Euroclone) containing 10% fetal bovine serum (FBS) (Gibco), 100 U/mL penicillin (Gibco), 100 mg/mL streptomycin (Gibco), and 2 mM glutamine (Gibco). The subcultivation ratio of 1 : 2 to 1 : 6 was generally applied.

### 2.4. Experimental Procedures

Cells were seeded in 10% FBS overnight; the following day media was removed and fresh 1% FBS AdipoRon-supplemented media was added to cell plates for times and concentrations indicated in the “Results” section. AdipoRon was prepared in DMSO. An identical amount (% v/v) of DMSO, named “untreated” in text and “NT” in figures, was used as the negative control.

### 2.5. MTT Assay

Cell viability was measured by the 3-(4,5-dimethylthiazol-2-yl)-2,5-diphenyltetrazolium bromide (MTT) assay. Briefly, 96-multiwell plates, consisting of 1.5 × 10^3^ cells/well (U2OS) and 2 × 10^3^ cells/well (Saos-2), were exposed for 72 h to increase AdipoR concentrations as described in the “Results” section. Subsequently, 100 *μ*L of MTT solution (5 mg/mL) was poured in each well and incubated for 3 hours at 37°C. Thereafter, media were removed and MTT-formazan crystals were solubilized in 100 *μ*L of isopropanol-HCl 0.04 N at room temperature on horizontal shaking for 30 minutes. Absorbance values were determined at 570 nm by using microplate reader. In every test, each single setting was replicated six times. MTT data such as media and standard deviation (SD) of at least three independent experiments were reported.

### 2.6. Cell Growth Evaluation

6-well plates, containing 7 × 10^4^ cells (U2OS) and 10^5^ cells (Saos-2), respectively, were employed to perform cell growth curve evaluation. In detail, cells were cultured with or without AdipoR for up to 72 h. After each experiment or each defined time point, cells were harvested using trypsin-EDTA solution 0.25% (Gibco) and spun down at 400 RCF for 5 minutes. Once resuspended in media, cells were counted in a Bürker chamber using an optical microscope. Average and SD values of at least three independent experiments were recorded.

### 2.7. Evaluation of Cell Cycle Distribution by PI-Staining

Cell cycle analysis was assessed by using a FACS-Calibur cytometer (BD Biosciences). Using the same experimental setting described in the previous subparagraph, pelleted cells were fixed in 70% ice-cold ethanol/PBS and incubated overnight at 4°C. The following day, cells were washed in PBS twice, spun down at 400 RCF for 5 minutes, and resuspended in 500 *μ*L of PI-staining solution (15 *μ*g/mL PI and 20 *μ*g RNaseA in PBS). Once incubated at room temperature for 10 minutes, protected from light, samples were analyzed by using a flow cytometer, and at least 50 K events were acquired. Lastly, percentage analysis of G1, S, and G2/M phases and subG1 events was calculated by using ModiFIT Cell Cycle Analysis software. Three independent experiments were performed.

### 2.8. Cell Extracts Preparation

A number of 6.0 × 10^5^ cells were seeded in 100 mm plates and treated with AdipoR for different times and concentrations. Then, the cells were collected, washed in PBS, and incubated on ice for 30 minutes in 3–5 volume of lysis buffer: RIPA buffer (R0278, Sigma-Aldrich), protease inhibitor cocktail (P8340, Sigma-Aldrich), and phosphatase inhibitor cocktail (P2850, Sigma-Aldrich). Later, samples were spun down at 18000 RCF for 15 minutes at 4°C. The supernatant (SDS total extract) was recovered, and protein content was determined by the Bradford Method. Finally, samples were diluted in Laemmli buffer 4X and boiled for 5 minutes at 95°C.

### 2.9. Western Blotting Analysis

Typically, 15 to 30 *μ*g of total cellular protein was loaded in polyacrylamide gel (Bio-Rad Laboratories) and separated by SDS-PAGE. Thereafter, proteins were transferred onto the nitrocellulose membrane (Sigma-Aldrich) using Mini Trans-Blot (Bio-Rad Laboratories). Before incubating overnight at 4°C using specific primary antibodies, membranes were blocked in no-fat milk (5% w/v) for 1 hour. The next day, horseradish peroxidase- (HRP-) conjugated goat anti-rabbit or anti-mouse antibodies were added to the membranes and kept for 1 hour at room temperature. TBS Tween-20 (Thermo Fisher Scientific) was used to wash the membranes (three times) before and after each incubation procedure. Enhanced chemiluminescence (ECL) (Euroclone) was employed to detect the HRP secondary antibody signal. To conclude, protein bands were detected with ChemiDoc XRS (Bio-Rad).

### 2.10. RNA Extraction and Real-Time Quantitative PCR

Total RNA was isolated from U2OS and Saos-2 cell lines, using TRIzol (Invitrogen, CA). Real-time quantitative PCR was carried out for 40 cycles at a melting temperature of 95°C for 15 sec and an annealing temperature of 60°C for 1 min. A dissociation curve was analyzed for each PCR experiment to assess primer-dimer formation or contamination. Relative mRNA level quantifications of target genes were determined using the cycle threshold method with GAPDH as a housekeeping gene, and the data were expressed as the expression relative to the housekeeping gene. The primers for AdipoR1, AdipoR2, CDH13, and GAPDH are available on request. The experiments were performed two times in triplicate.

### 2.11. Immunofluorescence Staining

U2OS and Saos-2 cells were cultured in 6-well chamber slides (Corning Incorporated, MA, USA) and fixed in 4% PFA for 20 minutes at room temperature. Slides were washed with and stored in PBS until staining. Cells were permeabilized and blocked in PBS containing 10% FBS and 0.01% Triton X (block buffer) for 1 hour at room temperature. Primary antibodies – AdipoR1 (H-001-44) and AdipoR2 (H-001-23) (Phoenix Pharmaceuticals, Burlingame, CA, USA) were incubated overnight in block buffer without Triton X at 4°C. After three 5-minute PBS washes, species-appropriate secondary antibodies (goat anti-rabbit IgG (H + L) cross-adsorbed secondary antibody, Alexa Fluor 568, Thermo Fisher, Waltham, MA, USA) were incubated at a 1 : 500 dilution in block buffer without Triton X for 2 hours at room temperature. Cells were washed in PBS, counterstained using DAPI (1 *μ*g/mL stock, 1 : 10,000 in PBS), and washed twice more in PBS. Images were acquired using a Zeiss LSM700 confocal microscope.

### 2.12. Statistical Analysis

Data were expressed as mean ± SD of biological replicates. Differences in mean between different groups were calculated by analysis of variance (ANOVA) and Student's *t*-test. Differences were recognized as statistically significant when *P* values are less than 0.05. Densitometric analyses were assessed using Image J 1.42Q (NIH, Bethesda).

## 3. Results

### 3.1. Adiponectin Receptors are Expressed in Saos-2 and U2OS Human Osteosarcoma Cells

**Figure 1:**
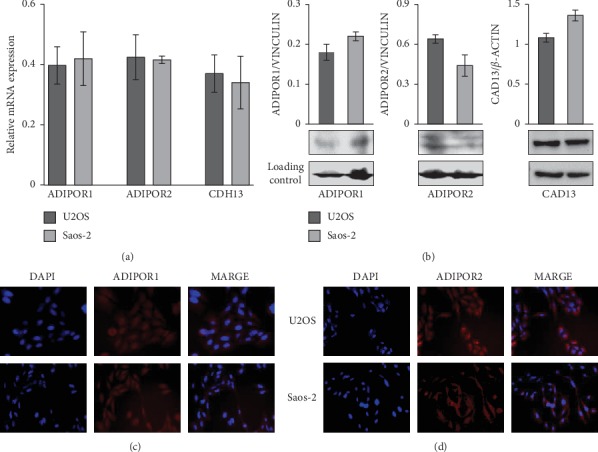
Evaluation of adiponectin receptors expression in U2OS and Saos-2 human osteosarcoma cell lines. (a) ADIPOR1, ADIPOR2, and CDH13 mRNA expression levels were determined by RT-PCR in *n* = 3 experiments. Data were standardized employing GAPDH and later quantified by 2^−ΔΔ*Ct*^ method. (b) Western blotting analyses were carried out to assess adiponectin receptors ADIPOR1, ADIPOR2, and CAD13 levels. *β*-ACTIN and VINCULIN were respectively used as internal loading control, and densitometric analysis was performed in order to normalize the data. (c and d) Immunofluorescence staining (red) for ADIPOR1 and ADIPOR2 were made in U2OS and Saos-2, DAPI staining (blue) was used for nucleus detection. Images were acquired applying a 20x magnification.

In order to explore the possible effects of AdipoR on human osteosarcoma cell behaviors, we first assessed the expression of adiponectin receptors in our experimental cell models. In detail, we detected in Saos-2 and U2OS human osteosarcoma cell lines mRNA and protein expression levels of both canonical adiponectin receptors (ADIPOR1 and ADIPOR2) and noncanonical adiponectin receptor (CAD13). According to previous findings [31], reverse transcription PCR (Figure 1(a)), immunoblotting (Figure 1(b)), and immunofluorescent analyses (Figures 1(c) and 1(d)) indicated that all evaluated adiponectin receptors were expressed in Saos-2 and U2OS, without significant variations between the two cell lines.

This evidence represents the preliminary remark for investigating the possible *in vitro* AdipoR antitumor effects in osteosarcoma.

### 3.2. AdipoRon Inhibit Proliferation in Saos-2 and U2OS Osteosarcoma Cells

To investigate whether adiponectin receptor agonist AdipoRon could affect the proliferation of human osteosarcoma cells, firstly we evaluated the consequences of AdipoR treatment on cell viability in Saos-2 and U2OS cells.

**Figure 2:**
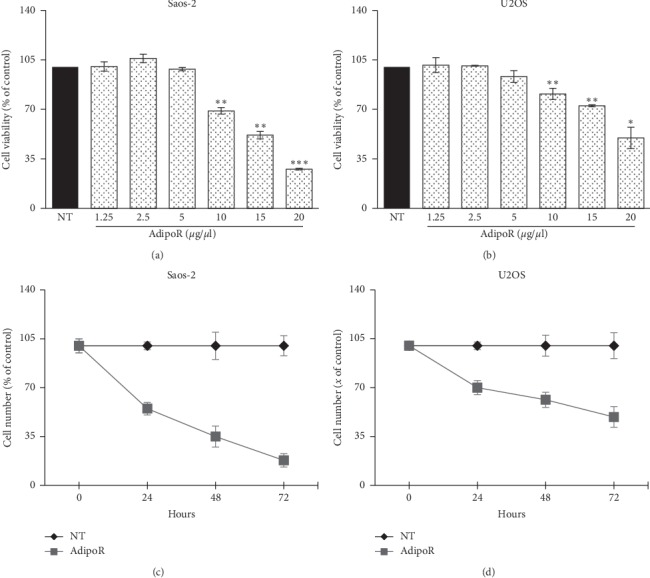
Effects of AdipoR treatment on the cell viability and cell growth in Saos-2 and U2OS cells. Saos-2 (a) and U2OS (b) were exposed to AdipoR (from 1.25 *μ*g/mL to 20 *μ*g/mL) for 72 hours in order to assess cell viability by MTT assay. Saos-2 (c) and U2OS (d) growth curves were defined after 24, 48 and 72 hours of treatment using 20 *μ*g/mL of AdipoR. Cell viability and cell growth curves were indicated in percentage of the counterpart “Not Treated” (NT). Mean and Standard Deviation (SD) of at least three independent experiments were reported. ^*∗*^*P* < 0.05, ^*∗∗*^*P* < 0.01, ^*∗∗∗*^*P* < 0.001 by unpaired *t*-test.

For this purpose, in agreement with previous studies [28–30], we treated Saos-2 and U2OS cells with a specific spectrum of AdipoR concentrations (from 1.25 *μ*g/mL to 20 *μ*g/mL) for 72 hours (h), and then MTT assays were performed. As indicated in Figures 2(a) and 2(b), AdipoR treatment up to 5 *μ*g/mL did not produce any significant variations in terms of cell viability in both cell lines analyzed, whereas 10 *μ*g/mL AdipoR provoked a reduction of 31% and 19% in Saos-2 and in U2OS, respectively. Notably, higher doses (15 *μ*g/mL and 20 *μ*g/mL) caused a clear dose-dependent cell growth inhibition in both cell lines, up to 50% in U2OS (Figure 2(b)), and even stronger, up to 72% in Saos-2 (Figure 2(a)).

Thereafter, time-course experiments have been also carried out. In detail, Saos-2 and U2OS were grown in the presence of 20 *μ*g/mL AdipoR for up to 72 hours; subsequently, cell number-counting data at 24, 48, and 72 h were employed to define relative cell growth curves.  Figure 2(c) shows that in Saos-2, the exposure to AdipoR leads to a drastic growth inhibition already after 24 h (45%), which becomes impressive at 48 and 72 hours, with a cell number decrease of 65% and 80%. In agreement with the abovementioned MTT results, a different extent has been obtained in U2OS, in which AdipoR caused a cell number reduction of 28%, 40%, and 50% after 24, 48, and 72 hours of treatment, respectively (Figure 2(d)).

Taken together, these results clearly demonstrate that AdipoR acts as an antiproliferative molecule both in Saos-2 and U2OS cells with different sensitivities between the two osteosarcoma cell lines, the former being more sensitive.

### 3.3. AdipoRon Induces G0/G1 Phase Accumulation and S Phase Decrease in Saos-2 and U2OS Cells

**Figure 3:**
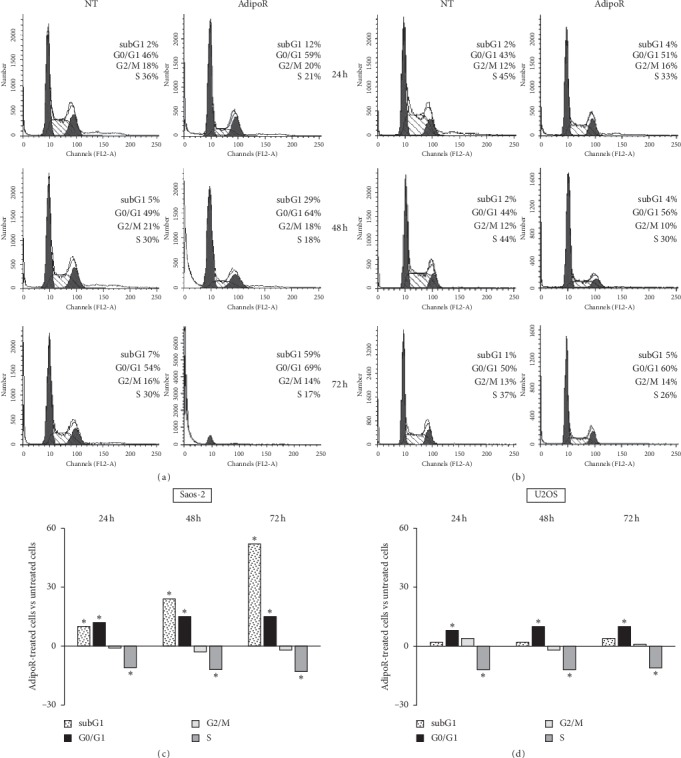
Effects of AdipoR treatment on the cell cycle distribution and subG1 phases in Saos-2 and U2OS cells. Saos-2 (a and c) and U2OS (b and d) were exposed to 20 *μ*g/mL of AdipoR for a time of 24, 48 and 72 h, and thereafter FACS analysis of PI-stained cells was performed. Representative FACS histograms of Saos-2 (a) and U2OS (b) cells exposed or not (NT) to AdipoR treatment were shown. Quantitative analyses of subG1, G1, S and G2/M percentage obtained from independent experiments performed in Saos-2 (c) and U2OS (d) were reported. Mean difference between AdipoR-treated cells and untreated cells has been used to chart the data. ^*∗*^*P* < 0.05 by unpaired *t*-test.

In order to further investigate and better explore the AdipoR-mediated antiproliferative effects in Saos-2 and U2OS osteosarcoma cells, we performed specific experiments aimed at assessing cell phase distribution in response to AdipoR treatment. Comprehensively, Saos-2 and U2OS were treated with 20 *μ*g/mL of AdipoR for up to 72 h, and thereafter, the consequences on cell cycle distribution were evaluated by flow cytometry using propidium iodide (PI) as a DNA-binding dye. Consistent with previous findings [32, 33] and most likely due to changes in cell density and nutrients availability occurred during time-course, the control-untreated cells are differently distributed in the cell cycle phases at the time points observed (Figures 3(a) and 3(b)). Regarding AdipoR-induced effects on Saos-2, Figures 3(a) and 3(c) display that the percentage of cells in the G0/G1 phase is significantly higher in AdipoR-treated cells compared with the untreated group. Simultaneously, a comparable decrease of the S phase is clearly observed at each time point analyzed (starting from 24 h and up to 72 h). An identical trend was observed in U2OS cells, where the consequences of AdipoR treatment on cell cycle distribution overlapped with those previously described in Saos-2 (Figures 3(b) and 3(d)).

Notably, an increasing time-dependent subG1 population (12%, 29%, and 59%, respectively, after 24, 48, and 72 h) is observed in Saos-2 cells in response to AdipoR (Figures 3(a) and 3(c)). As largely known, subG1 population represents the proportion of cells having a hypoploid DNA content, one of the biochemical hallmarks of apoptosis in which DNA begins to be fragmented. Interestingly, no obvious subG1 population is evident up to 72 h in U2OS in reaction to AdipoR (Figures 3(b) and 3(d)).

Altogether, the abovementioned data indicate that AdipoR causes a slowdown of the cell cycle division in Saos-2 and U2OS, promoting in the same time G0/G1 phase increase and S decrease. Moreover, in Saos-2 cells, but not in U2OS, AdipoR has a marked cytotoxic effect, suggesting a discrete and specific effect probably depending on the cell type and cellular genetic background.

### 3.4. AdipoRon Causes an Early ERK1/2 Activation in Saos-2 and U2OS Osteosarcoma Cells

Extracellular signal-regulated kinase (ERK) is the leading effector of a powerful intracellular signaling pathway capable of affecting nearly the totality of cellular functions, sometimes even radically opposite, such as cell growth and programmed cell death [34]. Considering its central role in cellular life, deregulation generally provokes the onset of several pathological conditions including carcinogenesis. Over the last decades, extensive studies have clearly indicated that the ERK pathway is relevantly connected to cancer cell proliferation in osteosarcoma [32, 35, 36]. Moreover, the involvement of ERK1/2 in both Acrp30 first and, more recently, AdipoR-mediated effects has also been also reported in tumor and nontumor cells [28, 37–40].

**Figure 4:**
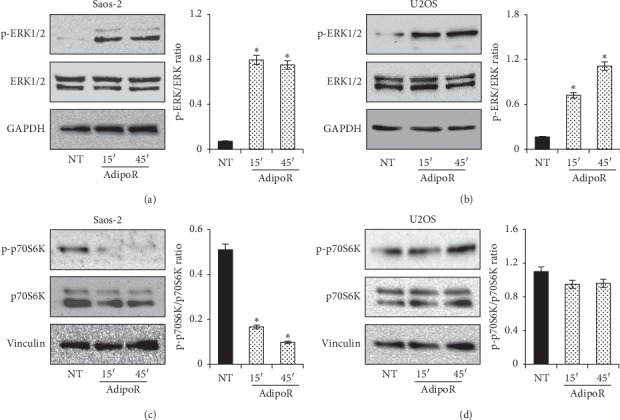
Involvement of ERK1/2 and p70S6K in AdipoR-mediated response in Saos-2 and U2OS cells. Immunoblotting of total and phosphorylated form of ERK1/2 and p70S6K were assessed after 15 and 45 minutes in response to 20 *μ*g/mL of AdipoR. Effects on total amount and phosphorylated levels of ERK1/2 in Saos-2 (a) and U2OS (b) were shown. Effects on p70S6K total and relative phosphorylated portion in Saos-2 (c) and U2OS (d) were reported. Densitometric analyses of phosphorylated/total ratio were also reported. ^*∗*^*P* < 0.05 by unpaired *t*-test.

To explore the possible implication of the ERK pathway in the AdipoR-induced antiproliferative effects in osteosarcoma cells, we studied the effects of AdipoR treatment on ERK1/2 activation/phosphorylation. Based on previous findings [37, 38], Saos-2 and U2OS were treated, for short times (15 and 45 minutes), with 20 *μ*g/mL of AdipoR, and subsequently total and phosphorylated forms of ERK1/2 were determined by immunoblotting. No variations were detected in the total amount of ERK1/2 in reaction to AdipoR in Saos-2, whereas a dramatic increase of ERK1/2 phosphorylation was observed after 15 and 45 minutes (Figure 4(a)). Data obtained in U2OS cells highlight a similar trend in ERK1/2 phosphorylation (Figure 4(b)).

Overall, these findings suggest that AdipoR generates a strong and immediate increase of ERK1/2 phosphorylation levels in both osteosarcoma cell lines, suggesting a possible involvement of this signaling pathway in AdipoR-mediated antiproliferative effects.

### 3.5. AdipoR Induces p70S6K Inactivation in Saos-2 but Not in U2OS Cells

The mechanistic target of rapamycin (mTOR) coordinates a multitude of eukaryotic cell processes mainly implicated in the modulation of cell growth and metabolism in reaction to specific external stimuli [41]. Adiponectin has been demonstrated to influence mTOR activity and, more specifically p70S6K, one of the best characterized downstream targets of mTOR complex-1 (mTORC1) [42–44]. Recently, even the aptitude of AdipoR to modulate p70S6K has also been addressed in preclinical models [30, 40].

In order to verify the possible involvement of p70S6K in AdipoR-mediated consequences in osteosarcoma cells, we checked total and phosphorylated levels of p70S6K in response to AdipoR treatment in Saos-2 and U2OS cells. Using the same experimental design, previously described for the assessment of ERK1/2 implication, we noted a dramatic decrease in p70S6K phosphorylation after 15 and 45 minutes in response to AdipoR in Saos-2 cells (Figure 4(c)). To reinforce the statistical significance of this data, no obvious variations were observed in the p70S6K total protein levels (Figure 4(c)). Surprisingly, AdipoR treatment did not provoke any alterations in both total and phosphorylated levels of p70S6K in U2OS (Figure 4(d)).

The above evidence indicates that AdipoR has a diversified and unequivocal effect on p70S6K activation in Saos-2 and U2OS cells, hypothesizing variances in its mechanism of action probably due to the different cell type on which it operates.

### 3.6. Everolimus-Mediated mTORC1 Perturbation Enhances AdipoR-Induced Effects in U2OS

AdipoR induced both antiproliferative and cytotoxic effects in Saos-2, whereas no cell death was found in U2OS. The analysis of two relevant cellular signaling pathways revealed a p70S6K dissimilar tendency to AdipoR treatment in these osteosarcoma cells. Therefore, we supposed a possible direct p70S6K involvement, alone or in the presence of ERK1/2 activation, in AdipoR-mediated cytotoxic effects.

**Figure 5:**
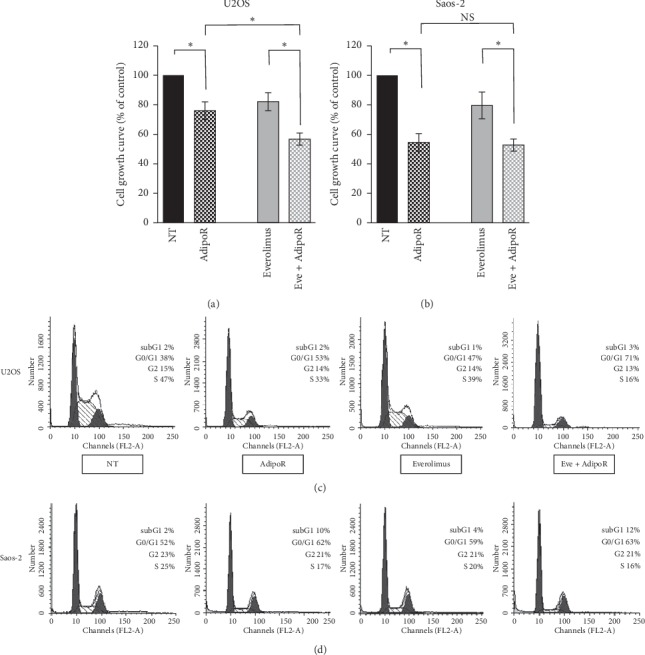
Effects of everolimus treatment on the AdipoR-induced cell growth inhibition and cell cycle phase assignment in Saos-2 and U2OS cells. Cells were treated with 20 *μ*g/mL of AdipoR, 50 nM of everolimus, and AdipoR plus everolimus for 24 hours, and then relative cell number was recorded (a and b) and cell cycle analysis (c and d) was performed. Specifically, panel a and c refer to U2OS, whereas panel b and d are related to Saos-2 cells. Mean and SD of three distinct cell number experiments were reported and expressed in % of control. Representative pictures of cell cycle phase distribution analysis were also displayed in panel (c) and (d) ^*∗*^*P* < 0.05 by unpaired *t*-test.

To address our speculation, we examined the impact of the combinatory treatment AdipoR/everolimus on cell proliferation and cell cycle distribution in U2OS cells (Figures 5(a) and 5(c)). Everolimus was chosen as the selective inhibitor of mTORC1, and 50 nM was used as active and broadly employed dosage [45, 46]. Cells were treated alone and in combination with AdipoR and everolimus for 24 h, and thereafter growth curves and cell phase distributions were assessed. According to our previous data, Figure 5(a) shows that in U2OS AdipoR causes a 26% reduction in terms of cell number, while a consistent impact on cell growth has also been detected in response to everolimus (−18% versus untreated group). Interestingly, the presence of both AdipoR and everolimus clearly enhances the single agent-mediated antiproliferative effects. In detail, we obtained the inhibition index of 54%, 22%, and 30% comparing cotreatment with untreated, AdipoR, and everolimus, respectively. Cell cycle distribution analyses of AdipoR- and everolimus-treated cells displayed a G0/G1 phase increase and a contemporary S decrease (Figure 5(c)). Although these two distinct treatments share a similar trend, the magnitude of this phenomenon is slightly different in AdipoR, where the numbers are more robust than in everolimus. Impressively, AdipoR/everolimus provokes a nearly G0/G1 block phase and a dramatic S phase depletion; however, as shown in Figure 5(c), no subG1 appearance has been observed. Analogous experiments, performed in Saos-2 simultaneously show no enhancement in AdipoR-induced antiproliferative effects mediated by everolimus on both parameters analyzed (Figures 5(b) and 5(d)).

Our findings indicate that everolimus-mediated mTORC1 perturbation enhances AdipoR-induced cell growth inhibition in U2OS and further slows down the cell cycle division without, however, significant cytotoxic effects.

## 4. Discussion

Despite the efforts made in the last decades to ameliorate its unfavorable prognosis, osteosarcoma still remains one of the most aggressive and metastatic tumors affecting children and teenagers [1, 2]. Therefore,looking for other innovative and more effective pharmacological strategies represents the unique weapon to fight this malignancy. Recently, the first oral adiponectin receptor agonist AdipoRon has been identified, but its potential pharmacological usage has marginally been explored [22–24]. In this regard, initial evidence candidates AdipoR as a potential antineoplastic molecule in various preclinical models, including pancreatic and ovarian cancer [28–30].

To date, no proof is currently available concerning the possible AdipoR antitumor effects in osteosarcoma. In this present study, we clearly demonstrate that AdipoR inhibits cell viability and cell growth in Saos-2 and U2OS osteosarcoma cell lines and that a gradual break in cell cycle progression is involved. Specifically, flow cytometric analysis of PI-stained cells reveals a robust G0/G1 phase accumulation and S decrease in response to AdipoR treatment. Our data are fully in agreement with two recent studies in which AdipoR has been reported to have anticancer properties affecting cell cycle distribution in a similar way [28, 30]. The perfect correspondence of existing and our current findings could suppose a precise mechanism of cell cycle control mediated by AdipoR in cancer cells; moreover, even though comprehensive experiments have never been performed, to address this hypothesis, it is important to keep in mind that even Acrp30 has been shown to mediate G1/S phase arrest in different cell models [38, 47].

Consistent with a different sensitivity observed in terms of cell viability and cell growth between Saos-2 and U2OS, we also detect a marked subG1 appearance in response to AdipoR only in Saos-2 cells. In this regard, the opposite p53 status of these two osteosarcoma models, wild-type in U2OS and null in Saos-2, might represent a reasonable explanation for the discrete AdipoR-mediated cytotoxic effects as well as for the dissimilar responsiveness observed between these two models. However, preliminary experiments performed in MG-63 osteosarcoma cells (p53 function deficient) suggest a mechanism of AdipoR-induced cell death that seems to be independent of p53 status (Figure S1a). Additionally, resistance to AdipoR has also been observed in this cell model compared to U2OS (Figures S1b and S1c). Comprehensive and more extensive studies are needed to clearly define p53 involvement in all AdipoR responses, anyway.

Although the contemporary occurrence of hypoploid subG1 population and caspase-3 activation (data not shown) suppose an apoptosis induction, we cannot rule out other apoptosis-independent mechanisms of cell death involved in AdipoR-mediated cytotoxic effects in Saos-2. In this regard, not completely exhaustive and partially controversial data are available concerning the relationship between AdipoR-induced cell death and apoptosis initiation. Messaggio et al. reported that AdipoR increases apoptotic positive cells in human and mouse pancreatic cancer [29], whereas Akimoto et al. demonstrate that AdipoR-treated MIA PaCa-2 cells die largely via RIPK1-dependent necroptosis and mitochondrial dysfunction-mediated autophagy [28].

The mechanistic target of rapamycin (mTOR) is a pivotal prosurvival signaling pathway that negatively affects autophagy and self-digesting independent cell death processes [41, 48]. Several evidence proves that AdipoR phosphorylates the upstream mTORC1 blocker AMP-activated protein kinase (AMPK); however, the resulting impact on mTORC1 still remains unpredictable [28–30, 42–44]. Our findings further confirm a not univocal and direct mTORC1 modulation mediated by AdipoR; indeed, while AdipoR causes a complete mTORC1 downstream target p70S6K inactivation in Saos-2, an unchanged trend has been detected in U2OS cells. The dissimilar tendency observed in p70S6K activation might explain, per se, the different AdipoR sensitivity in osteosarcoma cells and, in particular, the AdipoR-mediated cytotoxic effects occurred in Saos-2. Previous findings report that p70S6K directly modulates apoptosis through proapoptotic BAD and inhibitor of apoptosis (IAP) BIRC5 regulation [49, 50]. In addition, the involvement of p70S6K threonine 389 phosphorylation in autophagy has also been addressed [51, 52]. Therefore, we have employed mTORC1 inhibitor everolimus in order to verify the possible cell death induction in the presence of AdipoR in the U2OS model. Unfortunately, no subG1 appearance has been detected after 24 h of AdipoR/everolimus cotreatment; nevertheless, the enhancement of the antiproliferative effects detected in the presence of AdipoR/everolimus further supports the hypothesis of mTORC1 as an unrelated pathway in AdipoR-mediated responses in the U2OS cell model.

Across the years, a consistent number of studies have correlated the Acrp30-induced effects to ERK1/2 modulation and, in particular, to ERK1/2 activation [37–39]. Nevertheless, concerning the consequences of AdipoR treatment on ERK1/2, the emerging evidence does not define clear leanings. Akimoto et al. proved that ERK1/2 activation mediates AdipoR-induced cell death in MIAPaCa-2 [28], whereas Fairaq & colleagues display an attenuation of neointima formation in artery-injured mice after AdipoR administration via ERK1/2 downregulation [40]. Herein, we display a powerful ERK1/2 phosphorylation in both AdipoR-treated osteosarcoma cells that seems to mediate cell cycle slowdown on one side (U2OS), and both cell cycle brake and cell death on the other side (Saos-2). The existing evidence generally links ERK1/2 inactivation with a pronounced antiproliferative action in different preclinical cancer models, including in osteosarcoma [32, 36, 53]. However, in several cell models ERK1/2 signaling can also mediate cell cycle break and cell death induction [54–57]. Ussar and Voss clearly assert that ERK1/2 acts as a master regulator of G1 to S phase transition [55]; in particular, they show that while MEK1/ERK preferentially facilitates cell proliferation via cyclin D-CDK4/6, MEK2/ERK promotes G1/S growth arrest though p21cip1 recruitment. In support of the ERK1/2 operating role in cell death involvement, Cook & collaborators claim that ERK1/2 straight regulates mitochondrial membrane depolarization and antiapoptotic BCL2 protein [57].

Due to its common and fast activation, ERK1/2 may represent a possible AdipoR intracellular effector, thus representing a key factor to understand the mechanisms by which AdipoR produces its effects on osteosarcoma cell lines. For this purpose, investigating the ERK1/2 perturbation, alone and/or in combination with AdipoR, will constitute the next experimental stage in order to obtain additional information on designing the full schematic AdipoR models in osteosarcoma. The discrete AdipoR effects observed in these two osteosarcoma cell lines also represent, in our opinion, a very fascinating aspect that requires to be addressed separately and more in detail.

## 5. Conclusions

Our findings clearly indicate that AdipoR acts as an antiproliferative molecule in osteosarcoma cell lines, further supporting the emerging hypothesis of AdipoR as a novel anticancer compound. They represent the first important evidence in osteosarcoma model and we deeply believe that they should encourage the development and design of further AdipoR-based preclinical cancer studies. Over the last years, a considerable number of new possible molecules, including natural compounds, have been brought to light for their potential use in cancer therapy [58]; however, the viability of these pharmacological strategies still remains partially unknown. Thus, exploring the feasibility of AdipoR as an alternative method of healing in osteosarcoma could represent a future challenge by scientific community aimed to provide a new valid tool to overcome cancer.

## Data Availability

The data sets used and/or analyzed to support the current findings are available from the corresponding author on reasonable request.

## Supplementary Materials

Supplementary Materials Figure S1. Effects of AdipoR treatment on cell viability and cell cycle distribution in MG-63 cells. (a) MG-63 were treated for 48 hours with or without (NT) 20 *μ*g/mL of AdipoR, and thereafter FACS analysis of PI-stained cells was performed in order to define the relative cell cycle phases distribution. (b) MG-63 were exposed to increasing AdipoR concentration (from 1.25 *μ*g/mL to 20 *μ*g/mL) for 72 hours and then tested by MTT assay for mitochondrial activity content. (c) Illustrative image reporting a comparative analysis of cell viability data among Saos-2, U2OS, and MG-63 cells after 72 hours of exposure to 20 *μ*g/mL AdipoR. ^*∗*^*P* < 0.05, ^*∗∗*^*P* < 0.01 by unpaired *t*-test.
