# Supplementary material for: AdipoRon Affects Cell Cycle Progression and Inhibits Proliferation in Human Osteosarcoma Cells
Source: J Oncol. 2020 Jan 22;2020:7262479. doi: 10.1155/2020/7262479 (PMC7204133; doi:10.1155/2020/7262479)
Supplement: Supplementary Materials — Figure S1. Effects of AdipoR treatment on cell viability and cell cycle distribution in MG-63 cells. (a) MG-63 were treated for 48 hours with or without (NT) 20 μg/mL of AdipoR, and thereafter FACS analysis of PI-stained cells was performed in order to define the relative cell cycle phases distribution. (b) MG-63 were exposed to increasing AdipoR concentration (from 1.25 μg/mL to 20 μg/mL) for 72 hours and then tested by MTT assay for mitochondrial activity content. (c) Illustrative image reporting a comparative analysis of cell viability data among Saos-2, U2OS, and MG-63 cells after 72 hours of exposure to 20 μg/mL AdipoR. ∗P < 0.05, ∗∗P < 0.01 by unpaired t-test. [file 7262479.f1.pptx]

## Slide 1
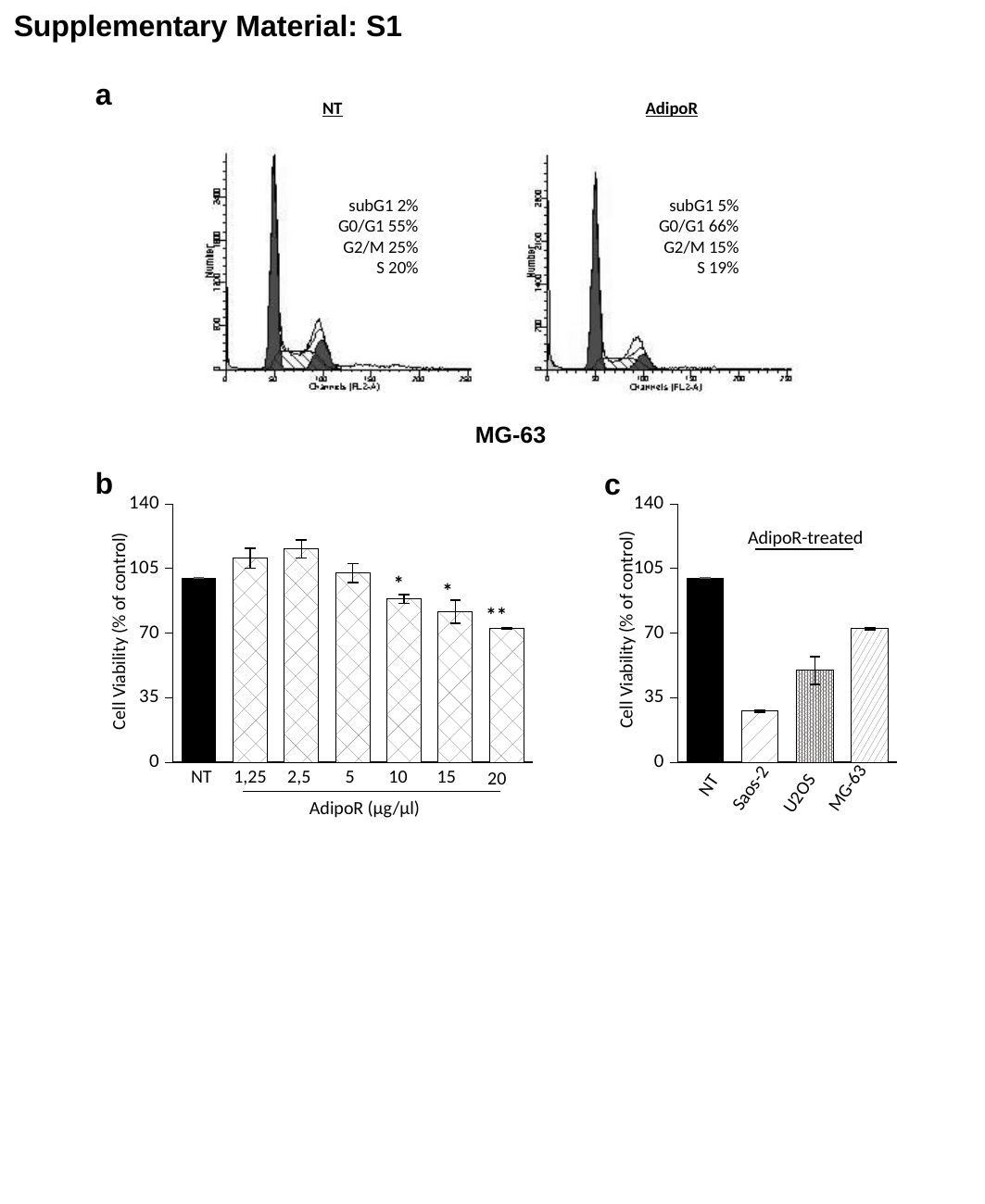

Supplementary Material: S1
a
AdipoR
NT
subG1 2%
G0/G1 55%
G2/M 25%
S 20%
subG1 5%
G0/G1 66%
G2/M 15%
S 19%
MG-63
b
c
### Chart
| Category | Serie 1 | Serie 2 | Serie 3 | Serie 4 | Serie 5 | Serie 6 | Serie 7 |
|---|---|---|---|---|---|---|---|
| Categoria 1 | 100.0 | 110.482784 | 115.64579999999998 | 102.48353 | 88.48353 | 81.60624 | 72.50834999999998 |Cell Viability (% of control)
5
10
NT
2,5
1,25
15
20
AdipoR (μg/μl)
*
*
**
### Chart
| Category | Serie 1 | Serie 2 | Serie 3 | Serie 4 |
|---|---|---|---|---|
| Categoria 1 | 100.0 | 27.73228999999999 | 49.79602000000001 | 72.50825 |AdipoR-treated
Cell Viability (% of control)
NT
Saos-2
MG-63
U2OS
